# Correction: Enhanced biohydrogen production from nutrient-free anaerobic fermentation medium with edible fungal pretreated rice straw

**DOI:** 10.1039/c8ra90061b

**Published:** 2018-07-18

**Authors:** Tao Sheng, Lei Zhao, Lingfang Gao, Wenzong Liu, Guofeng Wu, Jieting Wu, Aijie Wang

**Affiliations:** State Key Laboratory of Urban Water Resources and Environment, Harbin Institute of Technology Harbin 150090 China lzhao@hit.edu.cn +86 451 86282110; College of Environmental and Chemical Engineering, Heilongjiang University of Science & Technology Harbin China; CAS Key Laboratory of Environmental Biotechnology, Research Center for Eco-Environmental Sciences, Chinese Academy of Sciences Beijing China; Advanced Water Management Centre, Faculty of Engineering, Architecture and Information Technology, The University of Queensland Brisbane Queensland 4072 Australia; College of Life Sciences, Heilongjiang University Harbin China; School of Environmental Science, Liaoning University Shenyang China

## Abstract

Correction for ‘Enhanced biohydrogen production from nutrient-free anaerobic fermentation medium with edible fungal pretreated rice straw’ by Tao Sheng *et al.*, *RSC Adv.*, 2018, **8**, 22924–22930.

Affiliation *b* was incorrect in the published article and the corrected version is shown below.

The Royal Society of Chemistry apologises for these errors and any consequent inconvenience to authors and readers.

## Supplementary Material

